# Assessing the efficiency of China’s national fitness public services: a super-efficiency DEA-Malmquist–Tobit approach

**DOI:** 10.3389/fpubh.2024.1433337

**Published:** 2024-09-12

**Authors:** Xueting Gao, Li Cao, Qian Gu

**Affiliations:** ^1^School of Physical Education and Sport Science, Qufu Normal University, Qufu, China; ^2^School of Physical Education, Shandong Normal University, Jinan, China; ^3^School of Physical Education, Shandong University, Jinan, China

**Keywords:** national fitness public services, financial expenditure efficiency, super efficiency DEA model, Malmquist mode, Tobit model

## Abstract

**Introduction:**

As the Chinese government places an increasing emphasis on public fitness services, there has been a concomitant growth in public demand for greater fiscal expenditure in this area. However, in light of the constrained growth in government financial resources, it is of paramount importance to allocate these resources in a rational manner in order to effectively address the public’s fitness and health needs. This study aims to evaluate the efficiency of public expenditure on national fitness services across China, thereby providing valuable insights for policymakers to optimize resource allocation and improve service efficiency.

**Methods:**

The study employs a super-efficiency Data Envelopment Analysis (DEA) model, in conjunction with the Malmquist Index and Tobit regression model, to assess the efficiency of fiscal spending on fitness services in 31 Chinese provinces from 2017 to 2020. The analysis employs both static and dynamic approaches to present an objective view of the development of public fitness service levels across different regions and to empirically identify the key factors influencing fiscal spending efficiency.

**Results:**

The findings indicate substantial regional variations in the efficiency of fiscal expenditure on public fitness services. While some provinces demonstrate high efficiency in the use of public funds, others exhibit notable inefficiencies, particularly in areas with lower levels of economic development and population density. The findings underscore the existence of redundant expenditure and the varying effectiveness of resource utilization across provinces.

**Discussion:**

The study recommends that future strategies prioritize the scientific planning of fiscal inputs into public fitness services, the precise optimization of expenditure structures, the exploration of collaborative supply mechanisms, the expansion of demand-driven feedback channels, the integration of technological innovations, and the acceleration of digitalization in public fitness services.

## 1 Introduction

The comprehensive nationwide implementation of physical fitness activities represents a noteworthy innovation within China’s distinctive socialist framework (1). The 20th National Congress of the Communist Party of China explicitly delineated the objectives and responsibilities necessary to establish a modern socialist sports powerhouse, integrating physical fitness public services with pivotal national strategies. This role is of critical importance for the strategic advancement and historical mission of modernizing Chinese sports. The development of a balanced, scientific, and advanced physical fitness public service system not only fulfills the public’s aspirations for a healthy lifestyle but also provides essential support for the construction of a sports powerhouse and a healthier China. The promotion of universal, age-friendly physical fitness services on a national scale represents a pivotal step toward the achievement of modernization and shared prosperity through health investments.

By 2020, China had established a public fitness service system that was consistent with its objective of fostering a moderately prosperous society in all aspects. However, in light of the growing demand for fitness from the population, the inadequacy and imbalance in the provision of public fitness services have emerged as urgent issues that require immediate attention. Subsequently, the “Guiding Opinions on Building a Higher-Level Public Fitness Service System,” issued in 2022 by the General Office of the Communist Party of China and the General Office of the State Council, explicitly emphasized the crucial role of accelerating the modernization of public fitness service governance in developing a more sophisticated public fitness service system (2). The efficiency of public fitness service delivery, as measured by the ratio of national fiscal expenditure to the output of these services, is a critical indicator of the modernization of public fitness service governance. The focus on public expenditure efficiency represents a central theme in both international politics and research. Improving this efficiency is key to directing more resources toward public welfare (3). This is regarded as the optimal strategy for addressing the pressures of economic, demographic, and fiscal challenges (4, 5). As China continues to prioritize and invest in universal public fitness services, the scale of fiscal expenditures in this area has progressively increased. Nevertheless, a more comprehensive investigation is necessary within the academic community to gain a comprehensive understanding of the efficiency of these services, the factors influencing it, and the optimal strategies for utilizing limited financial resources to enhance service efficiency.

## 2 Literature review

The efficiency of financial expenditures on public fitness services serves as a pivotal indicator of the modernization of public fitness service governance. It elucidates the relationship between national financial expenditures and the output of these services. A comprehensive and objective evaluation of the supply efficiency of public fitness services across diverse Chinese provinces, cities, and autonomous regions, coupled with an in-depth examination of the factors influencing this efficiency, is vital for enhancing the quality and scope of public fitness services. The objective of these initiatives is to ensure the fulfillment of the fitness and health needs of the population. As the state places increased emphasis on the domain of sports public services, a multitude of policies and regulations have been enacted, attracting significant scholarly interest in topics such as the efficiency of sports public services and government performance evaluations.

Researchers have employed a variety of methodologies and models to facilitate comprehensive discussions and analyses. The extant body of research can be broadly categorized into three dimensions: In the initial stages of research, the focus was on developing an evaluation index system for sports public services. This objective was accomplished through the construction of index systems employing factor analysis, data envelopment analysis (DEA), the integration of the Balanced Scorecard (BSC) with the Analytic Hierarchy Process (AHP), Delphi development techniques, and radar analysis methods. These studies were followed by empirical studies (6–10). Subsequently, researchers employed multivariate statistical analysis methods to assess the levels of efficiency in the supply and expenditure of sports public services. The fundamental DEA model was utilized to assess the efficiency of sports public services or resource allocation at the provincial and municipal levels, indicating relatively low input–output efficiency (11–16). Moreover, innovative applications and methods have been documented, including the combination of DEA with exploratory spatial data analysis to evaluate efficiency output levels and spatial distribution (17–19), the integration of DEA with the Malmquist model for a comprehensive evaluation of financial inputs into sports public services (20, 21), and the employment of DEA and Tobit models to explore the factors influencing public sports services (22, 23). Furthermore, impact of alterations in relative technical efficiency on the efficacy of sports public expenditure in EU countries has been investigated (24). Additionally, Shao and Bo’s (2022) proposal of an intelligent evaluation method for public sports services based on the theory of intuitionistic fuzzy sets has been considered, which markedly enhances the reliability of these assessments (25). The DEA model has been extensively in a variety of sectors, including healthcare, where it has been utilized to assess the efficiency of hospitals and other healthcare providers. These studies underscore the model’s versatility and its capacity to accommodate a multitude of inputs and outputs, thereby rendering it a valuable instrument for assessing public service efficiency (26, 27). In the context of public fitness services, the application of DEA allows for a comprehensive evaluation of resource allocation and service delivery across different regions, which is crucial for understanding and addressing regional disparities. The DEA-Malmquist-Tobit model has gradually become a significant tool for measuring the combination of static and dynamic data and analyzing the influencing factors of panel data. The Malmquist index model has become a standard DEA method for measuring dynamic productivity due to its numerous advantages (28). The Tobit model is employed to address the issue of truncated data in efficiency evaluation, with the aim of estimating the contribution degree of variables to efficiency and subsequently analyzing the influencing factors (29). Previous studies have demonstrated the efficacy of integrating the Malmquist Index and Tobit models for the analysis of public service efficiency in diverse contexts, including healthcare and sports (30, 31). These studies have demonstrated the effectiveness of the models in capturing both the temporal dynamics of efficiency and the influence of external factors. By building on these methodologies, our study makes a contribution to the existing literature by applying this combined approach to the evaluation of public fitness services, offering new insights into the factors driving efficiency in this sector.

However, the majority of studies have concentrated on the fundamental, unchanging DEA model, which is unable to assess the efficiency of multiple units and disregards the dynamic evolution of performance trends. This paper diverges from the conventional focus on the fundamental DEA model and a static viewpoint by developing an evaluation metric for financial investment in public fitness services in China, utilizing provincial panel data from 2017 to 2020. The Super-Efficiency DEA-Malmquist-Tobit Model was selected for its capacity to account for regional heterogeneity in economic development and public service efficiency. This model permits a comprehensive examination of both static and dynamic efficiency, facilitating the identification of factors that contribute to efficiency variations across different provinces. The incorporation of the Tobit regression enables a more nuanced examination of the influence of these factors on efficiency scores, addressing the particular challenges posed by the heterogeneous economic contexts within China.

### 2.1 Theoretical foundation

It can be argued that public fitness services, which are crucial for satisfying the population’s fitness demands, are fundamentally characterized by their public welfare orientation. As the primary provider, the government ensures universal and equitable access to sports by offering a range of venues, facilities, events, fitness guidance, and other related services. In general, when a country’s *per capita* GDP reaches the moderately developed level, there is a greater propensity for fiscal expenditure to be allocated to public services such as public culture and social welfare (32). As the primary provider, the government bears the responsibility of ensuring that all citizens have access to universal and equal national fitness services, as well as the protection of their rights (33). In accordance with the public needs theory, the government is responsible for providing public goods in order to meet the needs of the public (34, 35). It is therefore expected that government-provided fitness services will be non-discriminatory, ensuring that all citizens are accorded equal access. However, due to the heterogeneity of inter-regional economic development in China, there are differences in financial expenditure and the total output of public fitness service supply for all. It is not feasible for all local governments to provide the same amount of public fitness services. Nevertheless, it is necessary to ensure the efficiency of financial expenditure, regardless of the amount, so that the financial expenditure of “less money, more work” can further improve the quality of national fitness public services. This theory provides a framework for evaluating the efficiency of public fitness services, emphasizing the importance of meeting public demand through effective and equitable resource allocation. In the context of this study, the theory is applied to evaluate the extent to which public fitness services align with these principles across different regions of China. However, the considerable heterogeneity in regional economic development across China has resulted in notable discrepancies in both the financial expenditure and the overall volume of public fitness services (21). Such disparities are influenced by a number of factors, including the allocation of funding for sports and the institutional frameworks in place. Moreover, there is an increasing public demand for more effective and comprehensive fitness services. It is therefore of the utmost importance to ensure the efficiency of financial expenditure. In order to significantly enhance the quality of public fitness services, it is essential to achieve the objective of “more with less.”

### 2.2 Research hypothesis

This study employs the Tobit model to identify the primary factors influencing the efficiency of fiscal expenditures on China’s national fitness public services, analyzing the impacts from social, economic, and cultural perspectives. In light of the extant literature and the distinctive characteristics of China’s national fitness public services, it is hypothesized that factors such as the level of economic development, population density, government size, and educational level significantly affect the efficiency of these fiscal expenditures. The specific hypotheses are outlined below.

*H1*: There is a positive correlation between the level of economic development and the efficiency of public expenditure on comprehensive fitness public services (15, 36–38). The analysis employs provincial gross domestic product (GDP) as a proxy for economic development. The majority of existing research indicates a positive correlation between economic development and service efficiency. Therefore, this study proposes that elevated levels of economic development enhance the government’s capacity to improve service efficiency. Nevertheless, the distribution of resources without regard for efficiency and the potential for wasteful expenditure could result in inefficiencies.

*H2*: There is a positive correlation between population density and the efficiency of public expenditure on comprehensive fitness public services. The number of individuals per square kilometer within each province serves as a proxy for population density. It is proposed that a higher population density may result in reduced costs associated with government management and supervision, thereby enhancing expenditure efficiency due to economies of scale (15, 39). Nevertheless, some academic perspectives propose a negative correlation between population density and service efficiency. These perspectives suggest that denser populations may result in overutilization and increased operational costs (37, 40). This hypothesis posits that a higher population density is associated with increased utilization rates of fitness services, which in turn facilitate resource access and reduce management costs.

*H3*: The greater the size of the government, the lower the efficiency of public expenditure on comprehensive fitness public services. n this study, the ratio of government consumption expenditure to GDP as a measure of government consumption scale. The relationship between government size and fiscal expenditure efficiency is a topic of contention in the academic literature. Marlow proposes that the efficacy of local government fiscal policies is inextricably linked to the congruence between public service supply and demand (41). Gordon, however, suggests that local government fiscal policies might result in external costs due to the potential for competitive rivalry among governments to reduce public service efficiency (42). Oates posits that fiscal policies exert a positive influence on the efficiency of public services. In the context of public cultural services, a larger government size is often associated with a reduction in the efficiency of fiscal expenditures (43, 44). Accordingly, the hypothesis put forth is that a larger government size impedes efficient fiscal management in public services.

*H4*: It is posited that the greater the level of education in a given region, the more efficacious the expenditure on comprehensive fitness public services. The hypothesis that the educational level, indicated by the proportion of the population with a specialized degree or above, is positively correlated with government financial efficiency (12, 45). However, some contest this correlation (37). This hypothesis posits that a higher proportion of the population with a university degree or above will result in greater awareness of sports activities, a higher demand for comprehensive fitness services, and an enhanced capacity for public engagement in governmental feedback and oversight. Consequently, this will result in an enhancement of governmental performance.

## 3 Materials and methods

### 3.1 Materials

#### 3.1.1 Evaluation index selection

The indicators selected for this study are primarily drawn from the “Sports Industry Statistical Yearbook,” a publication compiled by domestic scholars and the National Sports Bureau’s Economic Department. Based on an extensive review of the literature and data from the Economic Department of the General Administration of Sport of China, the Statistical Yearbook of China’s Sports Industry was compiled. This was combined with data from China’s national fitness public service to create a comprehensive study of 31 provinces, which were considered as decision-making units. The focus of the study was on the efficiency of financial expenditures on comprehensive fitness public services. In the context of comprehensive fitness public services in China, the outputs are closely related to factors such as the area of sports venues, the number of sports organizations and the availability of guidance services, as well as participation in mass sports activities (46). As comprehensive fitness public services are not itemized separately in financial expenditures, this paper employs findings from existing research to inform the selection of indicators based on data availability. The inputs and outputs selected for the DEA model were based on an extensive literature review and reflect the specific characteristics of public fitness services in China. The input indicators are comprised of two elements: *per capita* financial expenditure on comprehensive fitness public services (X1) (13, 22, 47) and the proportion of comprehensive fitness service expenditure within local financial budgets (X2) (13, 22, 36). The output indicators are as follows: the *per capita* area of sports venues (Y1) (13, 22, 48), the number of social sports instructors per 10,000 people (Y2) (11, 22), the number of sports social organizations per 10,000 people (Y3) (11, 13, 47), the number of mass sports activities conducted (Y4) (49, 50), and the proportion of the population regularly engaged in physical exercise (Y5) (11, 13, 15). The aforementioned indicators which are commonly used in similar studies to evaluate the efficiency of public services (11–16, 21, 37), and collectively constitute an evaluation index system for assessing the efficiency of comprehensive fitness public services, as shown in Table 1.

**Table 1:** Evaluation indicators.

Category	Indicator	Specific indicator	Definition	Unit
Input	Financial expenditure	*Per capita* financial expenditure on public fitness services (X1)	The total financial expenditure on sports-related services, including the public welfare fund from sports lotteries, divided by the total population of the region for the given year.	CNY (Chinese Yuan)
	Percentage of expenditure	Proportion of government’s fiscal expenditure on public fitness services (X2)	The ratio of the government’s expenditure on public services for national fitness, including funds from sports lotteries, to its total local fiscal expenditure for the year.	Percentage (%)
Output	Area of sports fields	*Per capita* area of public sports grounds(Y1)	The total area of sports facilities available in the region measured within the year.	Square meters (m^2^)
	Guidance services for sports organizations	Number of social sports instructors per 10,000 People (Y2)	The count of social sports instructors standardized per 10,000 inhabitants in the region.	Number
		Number of social sports organizations per 10,000 People (Y3)	The number of social sports organizations is standardized per 10,000 inhabitants of the region.	Number
	Mass sports participation	Number of mass sports activities conducted (Y4)	The total number of organized mass sports activities carried out in the region during the year.	Occurrences
		Proportion of the population regularly engaging in physical activity (Y5)	The percentage of the population that participates in regular physical activity relative to the total population in the area.	Percentage (%)

#### 3.1.2 Data sources

The data used in this study were mainly obtained from the “China Statistical Yearbook,” the “Sports Industry Statistical Yearbook,” and data provided by the National Bureau of Statistics, as well as the official websites of provincial sports bureaus (including municipalities and autonomous regions, but excluding Hong Kong, Macau, and Taiwan). The study covers the period from 2017 to 2020 and includes data from 31 provinces. Missing data were interpolated using the mean value method.

### 3.2 Methods

#### 3.2.1 Super efficiency DEA model

Data Envelopment Analysis (DEA) is a nonparametric method based on in linear programming that is used to assess relative efficiency (51). The traditional DEA models, specifically the CCR (52) and BCC (53) frameworks, serve as the foundation for this methodology. A number of scholars have extended these models to address a variety of research objectives, with the super-efficiency model representing a significant advancement. This model addresses the problem in conventional DEA frameworks where multiple Decision-Making Units (DMUs) are simultaneously considered efficient, each achieving an efficiency score of unity. This makes it difficult to make meaningful comparisons of their efficiency levels.

The input-oriented super-efficiency DEA model, proposed by Andersen and colleagues in 1993, differentiates DMUs by evaluating each unit in the context of an efficiency frontier formed by the other units, thus excluding the unit being evaluated (54). This approach allows efficiency scores to exceed unity, thus allowing differentiation through numerical comparisons between DMUs. It is worth noting that the efficiency scores of DMUs identified as inefficient remain unchanged, thus ensuring consistent assessments across the board. The super efficiency DEA model formula (55) is as follows:


$$\text{minθs}.t.\sum_{j\neq k^{j-1}}^{n}X_{j}\lambda_{j}\leq \theta X_{k}$$

$$\sum_{j\neq k^{j-1}}^{n}Y_{j}\lambda_{j}\geq Y_{k}$$

$$\lambda_{j}\geq 0,j=1,2,\cdots ,n\text{.}$$

Where *X* represents the input index, *Y* represents the output index, and *θ* represents the super-efficiency value. When we evaluate the relative efficiency of the *k*th DMU, the ideal output Y of the DMU can be expressed as all *k* DMU*j* except the input and output of the *k*th DMU (*j* = 1,2,…,*k*) of linear combinations.

**Figure 1:**
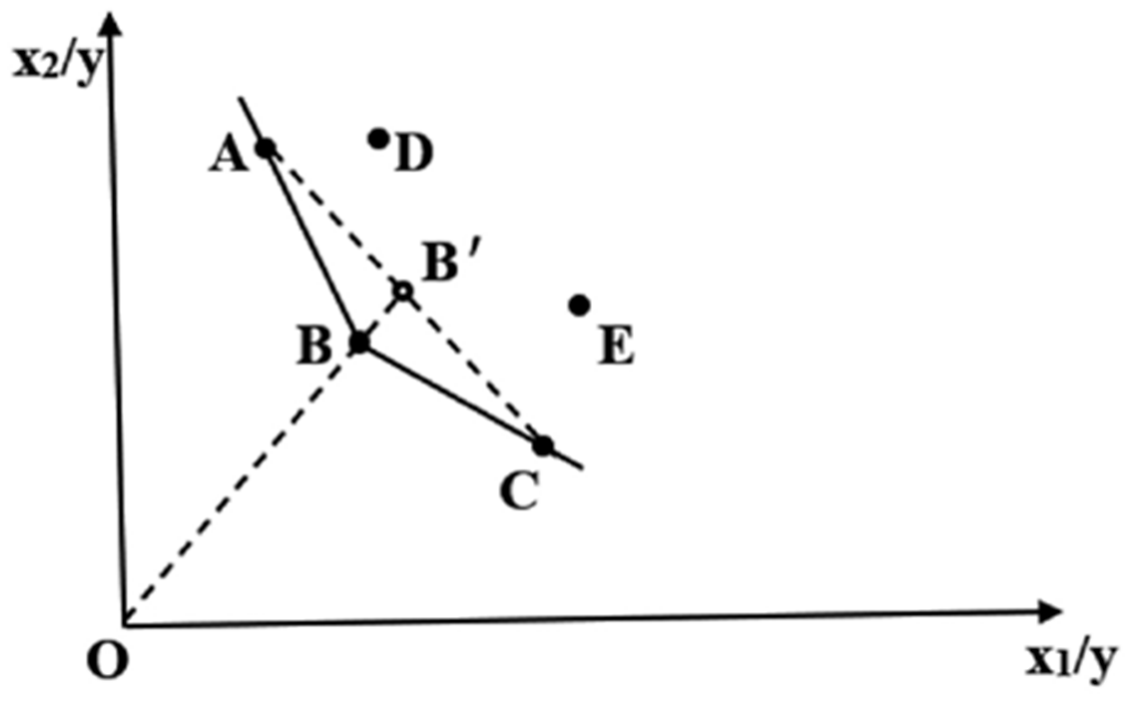
Schematic diagram of the super-efficiency model.

An illustrative example of this principle is the use of an input-oriented radial traditional CRS model. To illustrate, if DMUs A, B, and C are efficient and form the efficiency frontier, the analysis of DMU B would involve projecting it onto the frontier segment AC to determine point B′. The superior efficiency of B compared to B′ is quantified by the ratio of OB’ to OB, as shown in Figure 1.

#### 3.2.2 Malmquist index model

The concept of total factor productivity change (TFPC), which represents improvements in technological innovation and technical efficiency, was originally introduced by Solow (56). The Malmquist index model, developed from the DEA framework, is a well-known method for calculating TFP and has been extensively used in studies by Caves and others (57, 58). TFPC estimates are derived by using distance functions to quantify both Technical Efficiency Change (TEC) and Technical Progress Change (TPC), thereby elucidating the evolving dynamics in the provision of comprehensive public fitness services.

In this context, TPC represents the degree of technological advances or breakthroughs that occur between successive time periods. Conversely, TEC represents the effectiveness of resource allocation strategies. The decomposition of TEC into Pure Technical Efficiency Change (PTEC) and Scale Efficiency Change (SEC) is of paramount importance for a dynamic study of input–output efficiency. PTEC, by isolating the impact of scale, reflects adjustments in input intensity and provides insights into the administrative efficiency in delivering public fitness services. Conversely, SEC serves as a central metric for assessing whether changes in the scale of inputs are consistent with the optimal scale of production, thereby ensuring the scalability of resources.

In the Malmquist index model, the following symbols are employed to denote the distance function, input values, and output values, respectively, for a given time period:


$$\text{TFPC}=M\left(X_{t},Y_{t},X_{t+1},Y_{t+1}\right)$$

$$={\left(\frac{D_{t+1}\left(X_{t},Y_{t}\right)}{D_{t+1}\left(X_{t+1},Y_{t+1}\right)}\times \frac{D_{t}\left(X_{t},Y_{t}\right)}{D_{t}\left(X_{t+1},Y_{t+1}\right)}\right)}^{\frac{1}{2}}$$

$$=\frac{D_{t+1}\left(X_{t},Y_{t}\right)}{D_{t+1}\left(X_{t+1},Y_{t+1}\right)}\times {\left(\frac{D_{t+1}\left(X_{t+1},Y_{t+1}\right)}{D_{t}\left(X_{t+1},Y_{t+1}\right)}\times \frac{D_{t+1}\left(X_{t},Y_{t}\right)}{D_{t}\left(X_{t},Y_{t}\right)}\right)}^{\frac{1}{2}}$$

In the Malmquist index model, *D_t_*, *X_t_*, and *Y_t_* denote the distance function, input values, and output values, respectively, for a given time period *t*. The TFPC quantifies the change in productivity of a DMU from period t to *t* + 1. A TFPC value exceeding 1 signifies an enhancement in the level of supply, reflecting a favorable trajectory. Conversely, a TFPC value below 1 indicates a decline, suggesting a downward trend in the level of supply of national fitness public services. This metric is pivotal for evaluating temporal shifts in productivity and the adequacy of service supply in the context of national fitness services.

#### 3.2.3 Panel data Tobit model

The super-efficiency DEA framework allows the quantification of efficiency in the provision of national fitness public services across regions, resulting in discrete efficiency scores. Nevertheless, the model does not account for factors that are not directly related to inputs and outputs. Ordinary least squares (OLS) regression is the conventional tool for coefficient analysis. However, its applicability is constrained when confronted with discretely valued dependent variables, which can potentially lead to biased and inconsistent parameter estimates (59). To address this challenge, Tobin proposed a truncated regression model, replacing the ordinary least squares method with maximum likelihood estimation, known as the Tobit model (29). To address this gap, this study employs the Tobit model, which incorporates variables from social, economic, and cultural dimensions, with the aim of analyzing the main determinants affecting the efficiency of national fitness service supply. The Tobit theoretical model offers several advantages over other models. The model is designed to account for the inherent complexity of the national fitness public service system, which is predominantly government-supplied and encompasses a multitude of domains. This addresses issues related to singular inputs and multiple outputs. Secondly, the estimation methods employed in the Tobit model are designed to minimize bias. Finally, the Tobit model addresses truncation issues within the performance distribution, thereby facilitating a more accurate analysis of influential factors. Although the Tobit model is commonly used for the analysis of censored data, it is not without limitations in this context. This is particularly true given that the efficiency scores generated by the super-efficiency DEA model are not capped at 1. To address this issue, we conducted a series of diagnostic tests to ensure that the model’s underlying assumptions were met. Our findings indicated that the Tobit model was an appropriate choice for our analysis. Notwithstanding these limitations, the Tobit model remains an appropriate choice for examining the factors influencing efficiency scores in this study due to its capacity to accommodate the truncated nature of the dependent variable. Therefore, the efficiency score was selected as the dependent variable for the Tobit model because it directly reflects the relative efficiency of public fitness services across different provinces. In contrast to TFPC, which assesses changes over time, the efficiency score offers a more immediate evaluation of resource utilization, making it the most pertinent metric for our research objectives. This choice allows for a more precise analysis of the factors influencing current efficiency levels, which is central to the study’s aim of improving resource allocation in public fitness services.

### 3.3 Statistical analyses

The empirical work entailed the collection of panel data from 31 Chinese provinces over the period spanning 2017 to 2020. The inputs and outputs for the DEA model were carefully selected with great care, based on an analysis of existing literature and an examination of the specific characteristics of public fitness services in China. The initial phase of the statistical analysis is to verify that the collected data encompasses all DMUs relevant to the efficiency evaluation, thereby ensuring both completeness and accuracy. In order to facilitate the comparison of data sets with varying units and magnitudes, it is essential to standardize the data. Any missing values are interpolated using the mean value method. Moreover, to guarantee the rationality of the evaluation criteria, a correlation test is performed to confirm the positive correlation between input and output indicators, which is a prerequisite for the super-efficiency model. The initial evaluation of the static comprehensive efficiency of financial expenditures on comprehensive fitness public services is conducted using the Super Efficiency DEA model. Subsequently, the Malmquist index model is employed to assess the dynamic TFPC and its decomposition indicators, with a particular focus on the contributions of technological progress and efficiency changes to the overall productivity variation. Furthermore, Tobit regression was utilized to examine the variables affecting efficiency, considering the truncated nature of the efficiency scores. The following subsections provide detailed descriptions of the data sources, variable definitions, and model specifications.

## 4 Results and discussion

### 4.1 Efficiency measurement of fiscal expenditure on national fitness public services

#### 4.1.1 Static efficiency measurement based on the super efficiency model

##### 4.1.1.1 Correlation analysis among indicators

This stage involves the calculation of Pearson correlation coefficients, the results of which are presented in Table 2. The indicators demonstrate a significant correlation, thereby confirming the appropriateness of the selected evaluation metrics for the super-efficiency model.

**Table 2:** Correlation test between input indicators and output indicators.

Variables	X1	X2	Y1	Y2	Y3	Y4	Y5
X1	1						
X2	0.804**	1					
X3	0.184*	0.180*	1				
X4	0.079	0.092	0.345**	1			
X5	0.501**	0.486**	0.037	0.187*	1		
X6	0.755**	0.685**	0.295**	0.316**	0.622**	1	
X7	0.310**	0.407**	0.201*	0.357**	0.444**	0.568**	1

* Indicates *p* < 0.05; ** indicates *p* < 0.001.

##### 4.1.1.2 An analysis of the efficiency of financial expenditure on comprehensive fitness public services

The super-efficiency DEA model was applied to evaluate the efficiency of financial expenditure on comprehensive fitness public services at the provincial level in China. The results are presented in Table 3. The mean comprehensive efficiency score was found to be 0.62, indicating a general inefficiency (θ < 1) in the allocation and utilization of financial resources This highlights the urgent necessity for improvements in financial efficiency at the provincial level.

**Table 3:** Fiscal expenditure efficiency of national fitness public services in 2017–2020.

Province	2017	2018	2019	2020	Average
Beijing	0.33	0.30	0.25	0.21	0.27
Tianjin	0.30	0.27	0.25	0.27	0.27
Shanghai	1.11	1.05	1.06	1.09	1.08
Chongqing	1.07	1.04	1.03	0.57	0.93
Heilongjiang	0.50	0.42	0.52	0.54	0.50
Jilin	0.76	0.70	0.60	0.60	0.66
Liaoning	0.81	0.63	1.02	1.03	0.87
Hebei	0.36	0.31	0.36	0.38	0.35
Henan	0.76	0.64	0.69	0.67	0.69
Shandong	1.01	0.63	0.67	1.01	0.83
Shanxi	0.42	0.42	0.54	1.01	0.60
Shaanxi	0.46	0.48	0.45	0.53	0.48
Gansu	0.16	0.19	0.29	0.30	0.24
Qinghai	0.49	0.43	0.70	0.55	0.54
Sichuan	1.01	1.04	0.63	0.55	0.81
Hubei	0.52	0.39	0.39	0.44	0.43
Hunan	0.44	0.45	0.50	1.03	0.61
Jiangxi	1.01	0.54	0.47	1.03	0.76
Anhui	1.07	1.24	1.09	1.02	1.11
Jiangsu	1.22	1.04	1.14	1.26	1.17
Fujian	0.45	0.39	0.42	0.28	0.39
Zhejiang	1.06	1.16	1.09	1.05	1.09
Guangdong	0.73	1.01	0.68	0.70	0.78
Hainan	0.12	1.01	1.02	1.01	0.79
Yunnan	0.57	0.56	0.52	0.53	0.54
Guizhou	0.33	0.41	0.37	0.40	0.38
Inner Mongolia	0.38	0.40	0.40	0.42	0.40
Ningxia	0.20	0.21	0.20	0.19	0.20
Xinjiang	1.00	1.02	1.12	1.06	1.05
Guangxi	0.28	0.27	0.32	0.25	0.28
Xizang	0.19	0.17	0.20	0.20	0.19
Average	0.62	0.61	0.61	0.65	0.62

A comprehensive analysis of the data indicates a significant discrepancy in efficiency levels across provinces. It is noteworthy that five provinces—Shanghai, Jiangsu, Zhejiang, Anhui, and Xinjiang—achieved an efficiency rating of θ ≥ 1, with Jiangsu reporting the highest rating of θ = 1.17. These findings indicate that these provinces are effectively utilizing their fiscal resources. In contrast, the majority of provinces exhibited an efficiency score below 1, indicating that there is significant potential for improvement. Specifically, 34.5% of the provinces, including Chongqing and Jilin, exhibited efficiency values between 0.6 and 1 (0.6 ≤ θ < 1). It seems probable that this group represents regions where economic constraints necessitate relatively efficient fiscal management despite limited resources. Conversely, 48.4% of provinces, including major economic centers such as Beijing, Tianjin, and Hebei, exhibited efficiency values below 0.6 (θ < 0.6), indicating a classification of inefficiency. This inefficiency may be attributed to potentially lax management practices or an inefficient allocation of resources, which overshadow their high input–output ratios in comprehensive fitness public services.

Moreover, an examination of the data from multiple years reveals a complex trajectory. Notably, regions such as Tianjin and Hebei have exhibited a gradual improvement in efficiency, whereas Beijing and Chongqing have demonstrated a decline.

#### 4.1.2 Dynamic efficiency measurement based on the Malmquist index model

##### 4.1.2.1 Overall analysis

From a time series perspective, the Malmquist Index, representing the TFPC of financial expenditures on comprehensive fitness public services in China from 2017 to 2020, records an average value of 1.076 (see Table 4). The index indicates that the level of comprehensive fitness public services in China has exhibited an upward trend, with an average annual growth rate of 0.76%. It is noteworthy that the detailed metrics for the period from 2017 to 2020 indicate that the annual growth rate of PTEC is 0.16%, while SEC has experienced negative growth. This indicates that the expansion of TEC is predominantly attributable to enhancements in PTEC, rather than to increases in scale.

**Table 4:** Malmquist index of fiscal expenditure on national fitness public services in China from 2017 to 2020.

Range	TEC	TPC	PTEC	SEC	TFPC
2017–2018	0.926	1.125	0.945	0.980	1.042
2018–2019	1.087	0.963	1.067	1.019	1.048
2019–2020	1.029	1.110	1.040	0.989	1.143
Average	1.012	1.063	1.016	0.996	1.076

TEC, technical efficiency change; TPC, technical progress change; PTEC, pure technical efficiency change; SEC, scale efficiency change; TFPC, total factor productivity change.

The mean value and average annual growth rate of the Malmquist Index are 1.076 and 0.76%, respectively. While these figures indicate growth, they are relatively modest and underscore the substantial potential for enhancing efficiency of financial expenditures on comprehensive fitness public services in China. The period from 2017 to 2019 was distinguished by a fluctuating upward trend, with the primary influence being the negative growth in TPC. However, there was a notable positive increase in TPC from 2019 to 2020, indicating an encouraging upward trend and underscoring the impact of TPC on TFPC.

The observed trends in dynamic efficiency trends are closely associated with policy changes that occurred during the study period. For example, the observed increase in efficiency in certain provinces coincides with the implementation of targeted fiscal policies designed to enhance the delivery of public services. Conversely, the observed decline in efficiency in other regions may be attributed to policy shifts that resulted in reduced funding or a shift in the focus of public fitness programs. A detailed examination of these policy changes will facilitate a more nuanced understanding of their influence on efficiency and enable the identification of strategies for maintaining or enhancing service delivery in the future. In conclusion, the dynamic efficiency of financial expenditures on comprehensive fitness public services in China is influenced by both PTEC and TPC. This dual influence demonstrates the complexity of the factors at play and underscores the necessity for targeted strategies to enhance both the technical efficiency and technological progress underlying the provision of these services.

##### 4.1.2.2 Specific analysis

As detailed in Table 5, the decomposition of TFPC and its annual dynamic efficiency indicates that the TFPC values for 26 provinces exceed 1, accounting for 83.87% of the total. This suggests that there has been an improvement in TFPC across the majority of provinces. In contrast, the TFPC values for 15 provinces are below the mean, representing 48.38% of the total. This underscores the necessity for targeted efficiency enhancements in these areas.

**Table 5:** Malmquist index of fiscal expenditure on national fitness public services in various provinces from 2017 to 2020.

Province	TEC	TPC	PTEC	SEC	TFPC
Beijing	0.840	1.070	1.000	0.840	0.900
Tianjin	1.019	1.083	1.150	0.886	1.104
Shanghai	1.000	1.055	1.000	1.000	1.055
Chongqing	1.000	0.979	1.000	1.000	0.979
Heilongjiang	1.068	1.056	1.028	1.039	1.128
Jilin	0.996	1.057	0.962	1.035	1.053
Liaoning	1.001	1.086	1.000	1.001	1.087
Hebei	0.989	1.032	0.999	0.990	1.020
Henan	0.989	1.019	0.990	0.999	1.008
Shandong	1.000	1.019	1.000	1.000	1.019
Shanxi	1.193	1.064	1.185	1.006	1.269
Shaanxi	1.010	1.055	1.016	0.993	1.066
Gansu	1.311	1.086	1.282	1.023	1.424
Qinghai	1.066	1.071	1.069	0.997	1.142
Sichuan	0.908	1.018	0.915	0.993	0.925
Hubei	0.970	1.058	0.969	1.001	1.027
Hunan	1.086	1.044	1.083	1.002	1.133
Jiangxi	1.000	1.085	1.000	1.000	1.085
Anhui	1.000	1.051	1.000	1.000	1.051
Jiangsu	1.000	1.084	1.000	1.000	1.084
Fujian	0.873	1.040	0.867	1.007	0.908
Zhejiang	1.000	1.057	1.000	1.000	1.057
Guangdong	0.986	1.082	0.981	1.005	1.066
Hainan	1.029	1.194	1.000	1.029	1.229
Yunnan	0.943	1.040	0.948	0.995	0.981
Guizhou	1.041	1.066	1.039	1.003	1.110
Inner Mongolia	1.089	1.055	1.076	1.012	1.149
Ningxia	1.035	1.142	1.028	1.007	1.182
Xinjiang	1.016	1.144	1.000	1.016	1.162
Guangxi	0.963	1.016	0.971	0.992	0.978
Xizang	1.056	1.083	1.022	1.033	1.143
Average	1.012	1.063	1.016	0.996	1.076

TEC, technical efficiency change; TPC, technical progress change; PTEC, pure technical efficiency change; SEC, scale efficiency change; TFPC, total factor productivity change.

The annual growth rate of TFPC for comprehensive fitness public services stands at 0.76%. However, the growth of PTEC is relatively low and unstable, coupled with an annual negative growth in SEC of 0.4%. These figures indicate significant variations in TPC among provinces, underscoring the necessity for more precise investments in comprehensive fitness public services to promote technological advancement and enhance the scientific level of supply services. This is particularly important given the observed reduction in inputs and lower efficiency in resource allocation.

In terms of provincial performance, the TFPC for 15 provinces, including Hebei, Henan, Sichuan, Yunnan, and Guangxi, is below the average. This is attributable to diminished expenditure efficiency, which is predominantly shaped by PTEC and SEC challenges. Conversely, provinces such as Jilin, Hubei, and Guangdong are subject to the efficiency of TPC. Moreover, provinces such as Beijing, Tianjin, and Shaanxi are affected by an excessive scale, which has resulted in a reduction in efficiency. The considerable regional disparities in efficiency can be attributed to a number of factors, including differences in economic development, population density, and the policies enacted by local governments. For example, provinces such as Jiangsu and Zhejiang, which have higher economic growth rates, exhibit higher efficiency scores due to more effective resource allocation and more developed infrastructure. In contrast, provinces such as Gansu and Qinghai exhibit lower efficiency scores, which may be attributed to constrained financial resources and less efficacious governance structures. The case studies demonstrate the necessity for bespoke policy interventions that take into account the distinctive challenges faced by each region.

### 4.2 Analysis of factors influencing the efficiency of fiscal expenditure on national fitness public services

In accordance with the formulated research hypotheses regarding influencing factors, descriptive statistics were performed on the variable indicators, with the results presented in Table 6. The efficiency values derived from the super-efficiency DEA model exhibit a merging-sorting characteristic. To gain further insight into the efficiency of fiscal expenditure on national fitness public services in China, randomness tests were conducted using panel data, as illustrated in Table 3. The Hausman test was employed to ascertain the suitability of employing either a fixed effects model or a random effects model. Subsequent to the acceptance of the null hypothesis in the test results, the random effects model was adopted, thereby confirming that the foundational assumptions of this model were met. Subsequently, a panel data Tobit random effects model was implemented, structured as follows:


$$y_{\text{it}}=\alpha +\beta X_{\text{it}}+\mu_{i}+\xi_{\text{it}}\;\;i=1,2,\ldots ,n;t=1,2,\ldots ,T$$

In this context, the variable *i* represents the cross-sectional individual, while *t* represents time. *y_i_* represents the efficiency score *θ*, *α* is the constant term, *X_it_* denotes the variables of influencing factors, *β* is the coefficient of the influencing factor, *μi* is the random variable, and *ξ_it_* is the random disturbance term.

**Table 6:** Descriptive statistical analysis of variables.

Variable name	Mean	Standard deviation	Maximum	Minimum
Regional GDP	30148.967	24558.452	111151.60	1,349
Population density	461.456	706.506	3924.29	2.84
Ratio of government consumption expenditure	0.149	0.070	0.500	0.0697
Proportion of population with college degree or above	0.146	0.076	0.477	0.0643

To ensure the robustness of the Tobit model was robust, a unit root test was conducted, necessitating the application of logarithmic transformations to the variable data in order to address issues of heteroskedasticity. Two models were developed. Model 1 is a Tobit model that incorporates random effects, while Model 2 is a conventional random effects model that serves as a validation step following the exclusion of variables that were found to be insignificant in Model 1. The analytical results, as illustrated in Table 7, offer insights into the dynamics of the factors influencing the efficiency of fiscal expenditures.

**Table 7:** Tobit regression analysis of the efficiency of financial expenditure on comprehensive fitness public services from 2017 to 2020.

	Model 1		Model 2	
Variables	Ratio	*p*-values	Ratio	*p*-values
Regional GDP	0.0402	0.00000190	0.0266	0.00000203
Population density	0.00139	0.0000430	0.00137	0.0000804
Proportion of population with college degree or above	−0.518	0.705	−0.83	0.622
Percentage of population with tertiary education	−0.889	0.58	−0.75	0.681
Log-likelihood	−16.962579		−17.480463	
Rho value	0.6907944		0.6406089	

#### 4.2.1 Analysis of individual effect variance

The rho value, which represents the ratio of individual effect variance to total variance, indicates values of 0.690 and 0.640 for the two models, respectively. A comparison of these values with the log-likelihood values indicates that there is no significant difference between the models. This suggest that individual effects do not play a significant role in explaining the observed variations in efficiency across the 31 provinces and cities.

#### 4.2.2 Comparison of log-likelihood values

A comparison of the log-likelihood values between the two models reveals no significant differences, thereby confirming the effectiveness of both models in analyzing the efficiency factors. The results demonstrate that both models indicate a significant influence of regional GDP and population density on the efficiency of financial expenditure on comprehensive fitness public services (*p* < 0.01). These findings lend support to Hypotheses H1 and H2, indicating that higher levels of economic development and population density are positively correlated with service efficiency. This finding is consistent with those of previous studies and lends support to the theory that denser populations result in reduced input costs and enhanced efficiency (15, 39). However, in provinces or cities with high economic development or large population densities, variations in low efficiency may be attributed to excessive investment funds, which may result in redundancy and lower resource efficiency.

#### 4.2.3 Analysis of government consumption expenditure and educational attainment

Neither the share of government consumption expenditure nor the level of education has a significant impact on the efficiency of comprehensive public services, leading to the rejection of hypotheses H3 and H4. The lack of impact of government consumption expenditure on service efficiency may be due to administrative consumption control measures aimed at preventing excessive government size and resource waste. In addition, the representativeness of the educational attainment data from the National Bureau of Statistics may not fully reflect the educational level of the population, possibly contributing to the insignificant results.

## 5 Strength and limitations

The construction of a super-efficiency DEA-Malmquist model and the utilization of the Tobit model provide comprehensive insights into the fiscal expenditure efficiency of China’s national fitness public services. In particular, the Tobit model provides valuable quantitative guidance for policymakers through its marginal effect analysis, showing both positive and negative effects on fiscal efficiency.

While the Tobit model is instrumental in analyzing fiscal efficiency and dealing with censored dependent variables, it primarily facilitates correlation analysis and is limited in its ability to establish causality. The assumptions of the Tobit model may also simplify the complexities of the real world, making it difficult to deal with non-linear relationships or complicated interaction effects. These issues are critical for practical fiscal efficiency analysis and require further theoretical and empirical validation.

## 6 Conclusion and policy recommendations

### 6.1 Conclusion

The basic goal of constructing a higher level of national fitness public service supply and balance is to improve the efficiency of fiscal spending. In 2022, the General Office of the Central Committee of the Communist Party of China and the General Office of the State Council issued “Opinions on Building a Higher Level of National Fitness Public Service System.” This initiative aims to improve people’s physical fitness and operate a more efficient national fitness public service system. Based on the theoretical foundation of the super-efficiency DEA-Malmquist model, this study evaluates the efficiency of fiscal expenditure on national fitness public services in China’s 31 provinces from both static and dynamic perspectives and analyzes significant influencing factors such as economic development level and population density. Key findings from the empirical research using the Tobit random effects model include:

1. General Inefficiency: From 2017 to 2020, the efficiency of fiscal expenditure on national fitness public services averages 0.62, indicating a general inefficiency in which fiscal funds are not fully utilized. There is a notable disparity among provinces, with only five provinces achieving an effective status, led by Jiangsu Province with the highest efficiency score.
2. Dynamic Efficiency Trends: The Malmquist index shows an annual increasing trend in fiscal expenditure efficiency, albeit at a relatively low rate of 0.76%. The dynamic increase is mainly influenced by changes in PTEC and TPC.
3. Influencing Factors: Regional GDP and population density significantly influence the efficiency of fiscal expenditure, with both showing a strong positive correlation. This confirms the hypotheses that economic development and population density are crucial for improving service efficiency.

### 6.2 Policy recommendations

Based on the study’s conclusions, several policy recommendations are proposed to improve the efficiency of fiscal spending and the delivery of national public services:

1. Strategic Fiscal Planning: Align fiscal investment with the needs for sports development needs and increase the proportion of fiscal expenditure dedicated to national fitness within the sports industry. Adjust fiscal investment based on economic development and public demand for sports in different regions to promote equitable development among provinces.
2. Optimization of Fiscal Expenditure Structure: Address inefficiencies in the fiscal management of the sports industry by optimizing the structure of fiscal expenditure, improving the utilization of financial resources, and ensuring the effective allocation of national fitness resources.
3. Collaborative Supply Mechanisms: Enhance the participation of social capital in national fitness services through competitive mechanisms and modern resource allocation methods such as BOT (Build-Operate-Transfer) and PPP (Public-Private Partnerships), aiming to improve the PTEC and TPC of resource allocation.
4. Digital Development and Demand-Oriented Services: Accelerate the digital development of national fitness services, build an integrated national fitness smart sharing cloud platform, and establish a demand-oriented feedback mechanism to match the supply of fitness resources with the actual needs of the public.

By implementing these recommendations, the government can ensure the efficient and equitable operation of the national fitness public service system, ultimately leading to a healthier and more active population. Future research could extend the model used in this study to other public service sectors, such as health care or education, where similar efficiency challenges exist. In addition, examining the impact of specific policy interventions on efficiency in different regions could provide further insights into how to optimize public spending. These avenues of research would not only improve our understanding of public service efficiency, but also contribute to the development of more effective and equitable policy frameworks.

## Data availability statement

The original contributions presented in the study are included in the article/supplementary material, further inquiries can be directed to the corresponding author.

## Author contributions

XG: Writing – review & editing, Writing – original draft, Validation, Software, Methodology, Investigation, Formal analysis, Data curation, Conceptualization. LC: Writing – review & editing, Supervision, Resources, Conceptualization. QG: Validation, Writing – review & editing, Supervision, Methodology, Investigation, Formal analysis, Data curation, Conceptualization.
